# Development and Validation of a Liquid Chromatography-Mass Spectrometry Assay for Determination of Cromolyn Sodium in Skin Permeation Studies

**DOI:** 10.1155/2022/7437905

**Published:** 2022-04-21

**Authors:** Miranda Kay Holman, Stacy D Brown, Dorcas Frempong, Ashana Puri, Steven Dinh

**Affiliations:** ^1^Department of Biological Sciences, College of Arts and Sciences, East Tennessee State University, TN 37604, Johnson, USA; ^2^Department of Pharmaceutical Sciences, Bill Gatton College of Pharmacy, East Tennessee State University, TN 37604, Johnson, USA; ^3^College of Engineering and Computing, Florida International University, FL 33174, Miami, USA

## Abstract

Cromolyn sodium (CS) is a mast cell stabilizer administered to treat allergic diseases. A topical system would sustain its delivery and may be designed for treatment of atopic dermatitis. Established HPLC protocols for detection of CS are time consuming and intensive, indicating the need for a more streamlined method. This study aimed at developing and validating a sensitive and selective LC-MS method for quantifying CS in skin permeation studies that was less time and resource demanding. The optimized method involved an isocratic mobile phase (10 mM NH_4_HCO_3_, pH 8.0, 90% and ACN, 10%) at a flow rate of 0.25 mL/min. Detection involved direct MS/MS channels with m/z 467.0255 (precursor) and *m*/*z* 379.0517 (fragment) using argon as the collision gas. CS calibrants were prepared in PBS, pH 7.4, and methanol for validation (0.1–2.5 *μ*g/mL). To ensure no skin interference, dermatomed porcine skin was mounted on Franz diffusion cells that were analyzed after 24 h. The skin layers were also separated, extracted in methanol, and analyzed using the developed method. Retention time was 1.9 min and 4.1 min in methanol and buffer, respectively. No interfering peaks were observed from the receptor and skin extracts, and linearity was established between 0.1 and 2.5 *μ*g/mL. Interday and intraday accuracy and precision were within the acceptable limit of ±20% at the LLOQ and ±15% at other concentrations. Overall, the simplified, validated method showed sensitivity in detecting CS in skin without interference and was applied to demonstrate quantification of drug in skin following 4% cromolyn sodium gel exposure.

## 1. Introduction

Cromolyn sodium, disodium 5- [3-(2-carboxylato-4-oxochromen-5-yl)oxy-2-hydroxypropoxy]-4-oxochromene-2- carboxylate (CS), is a mast cell stabilizer that has been used for the management of allergic and exercise-induced asthma, systemic mastocytosis, and has also been reported to effectively prevent allergic reactions associated with atopic dermatitis [1–3]. It functions to inhibit histamine and leukotriene release that act as inflammatory mediators in an allergic response caused by antigen stimulation, as well as other nonspecific triggers like exercise [4]. CS also presents as a more attractive alternative to steroids, lacking the complications associated with them, such as gastric issues and decreased immunity [2].

CS therapy for asthma is administered intranasally or orally, which requires a multiple dose regimen due to its short half-life (∼80 minutes) and low bioavailability (∼1% orally and ∼7% intranasally), causing inconsistent use among patients [2]. These properties constitute the need for developing a transdermal product for CS in asthma patients to allow for extended release, decreasing the number of doses, thus increasing patient compliance [5]. Atopic dermatitis can cause sleeplessness and severe pruritus, and conventional therapies give less than satisfying results in relieving symptoms. Previous clinical studies have demonstrated promising results with CS as a topical agent for treatment, most notably because topical use allows for direct access to mast cells of the skin [3, 6]. However, no topical formulation containing CS is commercially available till date. Our studies are thus aimed at developing an effective gel incorporating CS for treatment of atopic dermatitis, utilizing skin as the mode for its delivery. In addition, the feasibility of transdermal delivery of CS for preventative therapy in asthma will also be explored.

The process of developing and optimizing any effective topical or transdermal product formula involves conducting in vitro skin permeation studies to assess the permeation profile of the drug from the prepared formulation matrices using diffusion cells of varied kinds. A prerequisite for successfully conducting skin permeation studies and obtaining meaningful data is to employ a selective and sensitive analytical tool to quantify the amount of drug in the different skin layers as well as in the receptor compartment of the diffusion cells. However, owing to the complexity of the skin composition and possibility of interference due to its components during an analytical estimation, developing such an analytical method for detection and quantification of the drug of interest can be challenging [7].

Systemic circulation and metabolism of pharmaceuticals has long been investigated, and analytical methods are developed for a wide range of use in drug detection. Some such methods investigate drug stability and separation from its degraded constituents, while others quantitatively assess drug composition of pharmaceutical formulations. These methods can also be adapted for use in human or model system biological fluids and tissues, although drug separation from inherent biological compounds should be investigated to ensure method accuracy. *In vitro* permeation studies are uniquely subjected to such requirement as direct analysis of drug diffusion through skin and do not implement extensive drug extraction techniques usually used for analysis of compounds in urine or serum [8]. As a result, analytical methods employed for such studies need to be able to selectively quantify target drug from skin components, like epidermal ceramides, that leach into permeation samples [9, 10].

Previous methods for quantifying amount of CS in receptor solution and skin have been developed but are either too time and resource intensive, do not extend to porcine skin use, or lack mention regarding selectivity in face of skin interference. There exist liquid chromatography-mass spectrometry (LC-MS) protocols for detecting CS in human plasma and urine [11–13], but none could be found for CS detection in the skin. High performance liquid chromatography (HPLC) methods have been developed and used for quantification of CS in cellophane [1] and synthetic membranes [14, 15] which are not capable of accurately mimicking permeation of drug via animal skin [1]. Of the studies that employ HPLC analysis on animal skin, such studies investigate permeation across hairless guinea pig [16, 17], hairless mouse [14], or rabbit skin [15], all of which have varying characteristics when compared to porcine skin. Differences in stratum corneum thicknesses, lipid composition, and the presence of hair can affect characteristics of permeation [18, 19]. A study by Rakesh and Anoop [2] utilized spectrophotometric methods on permeation across porcine ear skin but made no mention of any skin interference we observed during our application of various HPLC-UV methods. Finally, a method of HPLC coupled with ion pairing has been established for CS quantification [16, 17]. Ion pairing can pose its own issues, including an increased cost associated with the quantity of ion pairing agents and dedication of single columns for single agents, as well as an increased duration of analyses based on elevated complexity of the method, mobile phase, equilibration, and selectivity [20]. It appears then necessary to investigate the use of an LC-MS assay for detection and quantification of CS in porcine ear skin that allows for selective drug detection excluding interference and is time and resource efficient.

In the present study, a new simple, sensitive, and selective method for analyzing CS in skin permeation studies using LC-MS was developed. The present study is, thus, the first one that describes the development and validation of an LC-MS method for CS detection in skin with no interference due to the skin components.

## 2. Experimental

### 2.1. Chemicals and Reagents

CS, acetonitrile (ACN), ammonium hydroxide, and phosphate buffered saline, pH 7.4 (PBS 10X), were purchased from Fisher Scientific (Fair Lawn, NJ, USA). Ammonium hydrogen carbonate (NH_4_HCO_3_) and LC-MS grade water were procured from Millipore (Burlington, MA, USA) and Honeywell (Muskegon, MI, USA), respectively. Methanol was purchased from Concord Technology (Beichen, Tianjin, China). Deionized (DI) water was acquired from a Milli-Q water purification system (Millipore, Bedford, MA, USA).

### 2.2. Instrumentation

All LC-MS samples were run on a Shimadzu LCMS-IT-TOF instrument (Columbia, MD, USA) equipped with dual pumps (LC-20AD XR), temperature-controlled autosampler set at 4°C (SIL-20AC HT), in-line degasser (DGU- 20A3), and column oven (CTO-20A). The mass spectrometer utilized an electrospray (ESI) source operating in negative ion mode. The HPLC column used was an InfinityLab Poroshell HPH-C18 (2.1 × 150, 2.7 *μ*m) from Agilent Technologies (Santa Clara, CA, USA).

### 2.3. Preparation of Calibrants and Samples

CS calibrants were separately prepared in 1X PBS (10 mM, pH 7.4) and methanol. A solution of 1 mg/mL CS was initially prepared in these solvents and further diluted to prepare stock solutions of 100 *μ*g/mL. The following concentrations were then prepared from the stock solutions by dilution with the respective solvents: 0.1, 0.25, 0.5, 0.75, 1, and 2.5 *μ*g/mL. All calibrants were filtered through a 0.22-*μ*m nylon syringe filter membrane (New Oxford, PA, USA) before analysis.

To confirm the absence of interference from the skin components, porcine ear skin was dermatomed with dermatome 75 mm (Nouvag AG, Goldlach, Switzerland) to a thickness of ∼600 *μ*m and mounted on Franz Diffusion Cells (PermeGear, Hellertown, PA, USA) containing 1X PBS. After 24 h, the receptor solution was collected and analyzed by the optimized LC-MS method. Further, the stratum corneum was separated from the same skin sample by the technique of tape-stripping using D-Squame stripping discs (Dallas, TX, USA) [21]. Twenty adhesive tape discs were applied sequentially on the permeation area of the skin for 10 s using a constant force applicator. Tapes 1, 2, 3, 4, 5, 6–10, 11–15, and 16–20 were collected separately and placed in 5 mL methanol. The viable epidermis underlying the stratum corneum was scraped off with a pair of forceps and separated from the dermis layer. Viable epidermis and dermis were then manually minced and placed individually in 2 mL methanol. The tapes, epidermis, and dermis samples were allowed to shake at 200 rpm for 4 h (Orbi ShakerTM Jr. Benchmark, Edison, NJ, USA). Thereafter, the extracts obtained were filtered using 0.22 *μ*m nylon membrane syringe filters and analyzed using the developed LC-MS method.

### 2.4. LC-MS Method

The chromatographic separation involved an isocratic mobile phase (10 mM ammonium hydrogen carbonate, pH 8.0 and acetonitrile: 9 : 1) at a flow rate of 0.25 mL/min. The column was kept at 40°C. The mass spectrometer utilized liquid nitrogen as the nebulizing gas in the negative ESI source, which was maintained at 200°C. Detection utilized direct MS/MS channels with m/z 467.0255 (precursor) and m/z 379.0517 (fragment) with argon as the collision gas. Injection volume for all calibrants and study samples was 20 *μ*L.

### 2.5. Validation of LC-MS Method

Validation of the developed method was conducted following the International Council for Harmonization of Technical Requirements for Pharmaceuticals for Human Use guidelines for analytical procedures with respect to selectivity, linearity and range, limit of detection (LOD) and quantification (LOQ), and interday and intraday precision and accuracy [22]. As described in section 2.3., the in vitro skin permeation studies involve the use of 1X PBS in the receptor of the Franz cells and organic solvents such as methanol for the extraction of drug from the stratum corneum and other skin layers. As calibrants individually prepared in these solvents would be involved when quantifying the drug amount in the receptor and different skin layers, the developed LC-MS method was validated for the drug solutions prepared in the respective solvents, and the absence of skin interference and selectivity of the developed method was confirmed.

#### 2.5.1. Selectivity

Standard CS solutions prepared in PBS and methanol were evaluated to ensure no interference of endogenous skin components with the drug peak. Retention time (RT) and peak areas were taken into consideration to draw suitable inferences.

#### 2.5.2. Linearity and Range

The linearity range of calibration plots was confirmed and validated for the CS samples prepared in PBS as well as methanol over three days. For each matrix, a 6-point calibration curve covered the range of 0.1–2.5 *μ*g/ml, with specific points at 0.1, 0.25, 0.5, 0.75, 1.0, and 2.5 µg/ml. Least squares linear regression was applied, and goodness of fit was estimated based on the calculation of Pearson's determination coefficient (R^2^) for each plot.

#### 2.5.3. Intraday and Interday Accuracy and Precision

The developed method was assessed for interday accuracy, represented by (%) error, and interday precision, represented by (%) relative standard deviation (RSD), over 3 days for calibrants prepared in PBS as well as in methanol (range as specified in section 2.5.2). Similarly, intraday accuracy and precision were determined by analyzing and comparing replicates for all calibrants analyzed on the same day. For each day of validation, 6 replicate samples were prepared for each concentration. The peak areas were compared against a calibration curve for that day, and accuracy and reproducibility were calculated using these data. Acceptable (%) RSD and (%) error were assigned at ≤15%, or ≤20%, if at LOQ [23].

#### 2.5.4. Limit of Detection and Limit of Quantification

For determination of LOD and LOQ, signal-to-noise (s/n) ratio was taken into consideration, with LOD defined as approximate s/n ∼3 and LOQ as the lowest validated concentrations with (%) RSD and (%) error ≤20%.

#### 2.5.5. Method Application

In vitro permeation of CS from a topical gel formulation (*n* = 3) through dermatomed porcine ear skin was studied employing the Franz Diffusion cell set up and quantifying the drug in the different skin layers using the optimized LC-MS method. Cells with 0.64 cm^2^ as the diffusion area were used. Skin was clamped between the donor and receptor compartment of the cells, such that stratum corneum layer faced upwards away from the receptor, and its temperature was maintained around 32°C. The receptor chamber consisted of 1X PBS, and the donor formulation was 100 *μ*L of CS gel (4% w/w drug in propylene glycol with 1% w/w hydroxypropyl cellulose as gelling agent). After 24 h, the unabsorbed gel was removed from the skin using Kim wipes, and the skin surface was washed sequentially three times, each with lauryl ether sulfate and DI water. CS retained in the stratum corneum, underlying viable epidermis, and dermis was extracted using methanol following a similar protocol as described in section 2.3 and quantified using the validated LC-MS method.

## 3. Results and Discussion

### 3.1. Method Development

**Figure 1:**
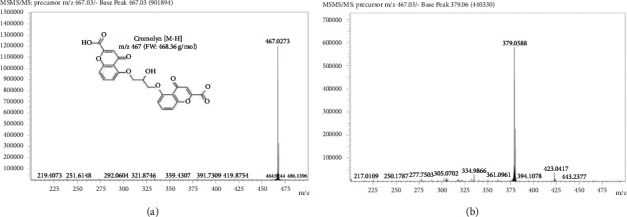
Negative electrospray mass spectra showing a base peak of (a) 467 *m*/*z* for CS in the precursor (MS1), as well as the chemical composition of CS and (b) 379 *m*/*z* for CS in the fragment (MS2). Abbreviations: CS, cromolyn sodium; *m*/*z*, mass to charge ratio.

Our initial method development focused on replicating the conditions of Lin et al., using reversed phase conditions and 10 mM ammonium acetate with 0.02% trifluoroacetic acid and methanol in a gradient [13]. These conditions worked well with aqueous samples, but significant ion suppression using our instrumentation was noted with samples in PBS or methanolic skin extracts. Furthermore, these conditions promoted the intermittent formation of sodium adducts, as noted by Lin et al. [10]. Previously published LC-MS assays for CS quantification relied on ionization by positive ESI [11–13], but the structure of this drug does not lend to easy ionization in positive mode. As noted in Figure 1(a), the free acid form of this drug contains two carboxylic acid groups, which would readily ionize in negative mode. As such, our method was built around the exploitation of the natural tendency for negative ion formation. The precursor *m*/*z* 467 fragments to *m*/*z* 379 have been shown in Figure 1(b). Solubility of the CS salt as well as negative ion formation can be facilitated by higher pH conditions; thus, an HPH-C_18_ column was chosen for the separation over a traditional C_18_. Buffer strength was optimized at 10 mM with a pH of 8.0. Finally, an isocratic separation was chosen over gradient to promote sample throughput.

### 3.2. Method Validation

**Figure 2:**
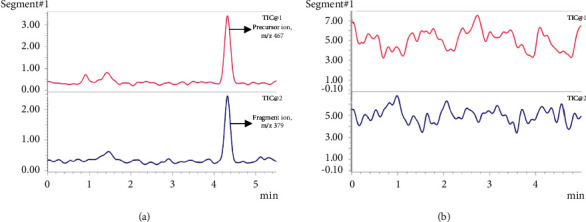
Chromatogram showing (a) pure 0.1 *μ*g/mL CS in PBS (b) receptor solution of PBS in the blank in vitro permeation study after 24 h set up with TIC1 = m/x 467 and TIC2 = *m*/*z* 379 Abbreviations: CS, cromolyn sodium; PBS, phosphate buffered saline; TIC, total ion chromatogram.

**Figure 3:**
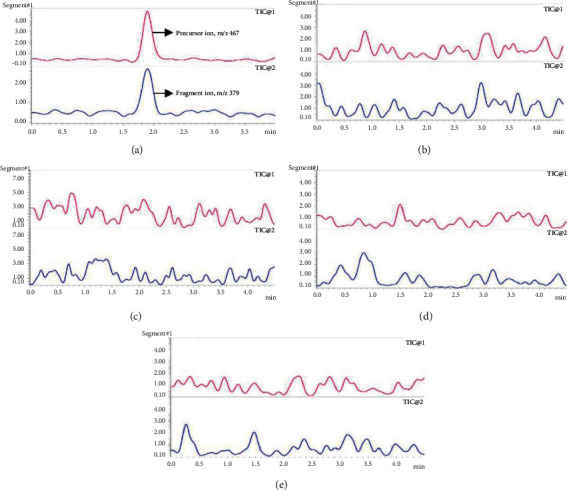
Chromatogram showing (a) pure 0.1 *μ*g/mL CS in methanol ((b)-(e)) skin extracts and matrices in methanol based on the blank in vitro permeation study set up with TIC1 = m/x 467 and TIC2 = *m*/*z* 379 ((b) and (c)) tape 1 and tapes 16–20 representing the stratum corneum; (d) viable epidermis extract; (e) dermis extract. Abbreviations: CS: cromolyn sodium; TIC, total ion chromatogram.

Example chromatograms for CS in PBS and methanol are shown in Figures 2(a) and 3(a), respectively. Differences in CS retention time can be accounted for by differences in solvent strength, with CS identity confirmed by the presence of *m*/*z* 467 ⟶ 379 transition. Various blank matrix extracts (receptor, stratum corneum, viable epidermis, and dermis) as described in Section 2.3 were analyzed, using the MS1 and MS2 channels associated with CS. Based on the extracted ion chromatograms at the precursor and fragment ions (*m*/*z* 467 and 379, respectively), no interfering peaks were found in any of the matrices (see Figure 2(b) for blank receptor PBS, and 3B-E for blank stratum corneum tapes, viable epidermis, and dermis extracts in methanol). The method shows good linearity across the calibration range in both PBS and methanol, as defined by Pearson's coefficient (R^2^) > 0.99. More specifically, the average R^2^ for triplicate PBS and methanol calibrations was 1.0 ± 0.00 and 0.9971 ± 0.0019, respectively.

The data for the method's intraday and interday accuracy and precision evaluation for PBS and methanol samples are summarized in Tables 1 and 2, respectively. Both intraday and interday precision and accuracy, reflected as (%) RSD and (%) error, are within the predetermined acceptable ranges in both PBS and methanol. Of note, the (%) RSD and (%) error was <15% for all concentrations except the LOQ (0.1 *μ*g/mL), which fell below 20%. Additionally, the LOD, based on a signal-to-noise ratio of 3 : 1, was 0.05 *μ*g/mL.

### 3.3. Method Application

**Figure 4:**
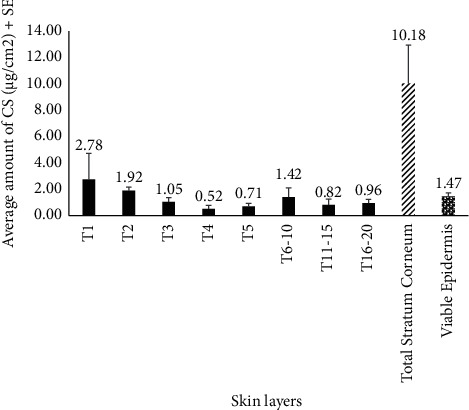
Distribution of CS (mean ± SE) in different skin layers, post-application of a 4% topical gel (*n* = 3). “T1- T20” refers to the different tapes, sequentially used to remove the stratum corneum. Abbreviations: CS, cromolyn sodium; SE, standard error.

Following the validated LC-MS method, total average amount of CS found in the stratum corneum and viable epidermis was calculated to be 10.18 ± 2.94 *μ*g/cm^2^ and 1.47 ± 0.27 *μ*g/cm^2^ respectively, and negligible drug retention (<LOQ) was observed in the dermis. More detailed distribution of the drug amounts in the individual tapes (representing the different layers of stratum corneum), entire stratum corneum (summation of the amounts in all the tapes), and viable epidermis are shown in Figure 4. Owing to CS's physicochemical properties such as high molecular weight (512.33 Da), hydrophilicity, and ionizability (pka of 1.9), its absence or extremely low amounts in the dermis is justified and highlights and supports the need for enhancers to increase delivery and permeation of CS in transdermal skin studies [2, 24, 25].

### 3.4. Method Justification

There exist various methodologies already established and validated for the detection of CS in pharmaceutical formulations or biological fluids, as summarized in Table 3. Some methods were designed and validated for CS detection in commonly prescribed nasal or ophthalmic pharmaceutical formulations, with interest in developing stability-indicating methods designed to separate CS from its degradation products [26–29], coadministered drugs [1, 30, 31], or both [32]. Few methods were only concerned with quantitation of CS present in formulation [33–36]. Some of these methods lack sufficient sensitivity for detecting lower quantities of CS present in skin permeation studies regarding earlier receptor time points and drug extracted from collected tapes or skin layers and would not provide accurate results. On the other hand, some of these methods had a small range of validated concentrations, which would not be ideal for CS determination in large quantities (that can also be observed in receptor solutions or skin layers) without dilution, making it a time and resource intensive analysis. Other spectroscopic [37] and HPLC-UV [38] methodologies designed to quantitate CS levels in formulation are described, but not much information is available on their development and validation.

Additional works have presented methods for determining CS quantity in biological fluids like aqueous humor, urine, serum, or plasma, often supporting their validation by confirming drug content from stock solutions or formulations. Moreira et al. were able to establish an effective method for trace detection of CS, but the authors concluded their approach would not be ideal for detection where CS is the primary component due to their method's high variation in accuracy and precision [39], such as for use in in vitro permeation studies evaluating CS delivery. Similarly, some of these methods used on equine urine [40], human serum [41], or human plasma [42] focus on quantitation of smaller amounts of CS, where method validation does not extend to high enough drug concentrations for convenient usage in permeation experiments without having to dilute the large number of samples. For methods where sensitivity is appropriate for permeation study application, added steps are required for analysis. Leavitt et al. [33], Gardner [43], and Aswania et al. [44] use protocols that employ more extensive separation, extraction, and purification methods that would not only be difficult for use on skin layers but are more time and resource intensive than the method reported by us.

Importantly, none of the discussed methods detailed in Table 3, regardless of sensitivity, have been evaluated for their use on skin and do not investigate selectivity of their methodology for CS detection against skin component interference either. Endogenous compounds from skin, such as lipids, leach into in vitro permeation study samples and are difficult to remove. For this reason, methods for drug detection in skin need to be selective in how they separate drug from these contaminants. A recent publication by Mulabagal et al. demonstrated the validation of an HPLC-UV method for quantitation of acyclovir and lidocaine in topical formulations for use in in vitro permeation studies through human cadaver skin [45], showing successful drug separation from skin leachables. While there are methods that have been validated for use in permeation studies, only two have been developed for CS detection in skin. An ion-paired HPLC method has been used in iontophoretic CS delivery across hairless guinea pig skin but has not been evaluated for quantitating drug selectively in porcine ear skin and from its interfering skin components [16, 17]. Ion-paired HPLC methods are also more time and resource consuming [20], indicating the need for a simpler method. A spectrophotometric method has also been used to detect CS content in in vitro permeations studies across porcine ear skin but does not establish its validated parameters and lacks mention and investigation of CS separation from skin interference [2]. The simple validated method reported in this paper is then the first to quantify CS sensitively and selectively from endogenous skin components and can easily be implemented for in vitro permeation studies employing porcine ear skin.

## 4. Conclusions

A negative mode ESI-LC-MS assay for quantification of CS in PBS and methanol has been developed and validated. The precision and accuracy for all points in the calibration curve, in the range of 0.1–2.5 *μ*g/mL, fell within acceptable limits (<15%; <20% at LOQ) for both intraday and interday experiments. The use of the mass spectrometric detection helps eliminate interference from the skin matrix by monitoring direct channels associated with the precursor (*m*/*z* 467) and major fragment ion (*m*/*z* 379). Use of the HPH-C_18_ column allows for higher buffer pH conditions than a conventional C_18_ column, helping promote ESI conditions conducive for the acidic cromolyn analyte. Finally, the method was successfully applied to investigate the permeation of CS into the skin from a topical gel. Further, the validated method would find application in studies investigating the percutaneous permeation of CS from different topical and transdermal formulation matrices, designed with the perspective of enhancing its permeation, into and across skin.

## Figures and Tables

**Table 1:** Intraday and interday accuracy and precision for CS in 1X PBS.

	Nominal concentration (*μ*g/mL)					
	0.1	0.25	0.5	0.75	1	2.5
*Intraday*						
Day 1 Mean (*N* = 6)	0.095	0.243	0.481	0.724	1.074	2.483
SD	0.016	0.024	0.061	0.072	0.117	0.215
Accuracy (% error)	4.632	2.904	3.732	3.497	7.378	0.680
Precision (% RSD)	17.1	9.743	12.701	9.914	10.937	8.657
Day 2 Mean (*N* = 6)	0.094	0.221	0.498	0.761	1.042	2.483
SD	0.016	0.028	0.064	0.082	0.073	0.141
Accuracy (% error)	**5.946**	**11.485**	**0.342**	**1.458**	**4.21**	**0.667**
Precision (% RSD)	**17.531**	**12.482**	**12.907**	**10.81**	**6.97**	**5.668**
Day 3 Mean (*N* = 6)	0.112	0.282	0.473	0.732	0.989	2.511
SD	0.022	0.02	0.049	0.105	0.143	0.158
Accuracy (% error)	**12.109**	**12.964**	**5.347**	**2.466**	**1.077**	**0.459**
Precision (% RSD)	**19.442**	**6.979**	**10.297**	**14.368**	**14.415**	**6.306**

*Interday*						
Average concentration (*μ*g/mL)	0.101	0.249	0.484	0.739	1.035	2.493
SD	0.019	0.034	0.056	0.084	0.113	0.164
Accuracy (% error)	**15.989**	**11.511**	**10.18**	**9.201**	**8.34**	**4.965**
Precision (% RSD)	**19.123**	**13.817**	**11.551**	**11.345**	**10.963**	**6.590**

**Table 2:** Intraday and interday accuracy and precision for CS in methanol.

	Nominal concentration (*μ*g/mL)					
	0.1	0.25	0.5	0.75	1	2.5
*Intraday*						
Day 1 Mean (*N* = 6)	0.114	0.276	0.511	0.660	1.030	2.510
SD	0.020	0.033	0.049	0.038	0.100	0.152
Accuracy (% error)	**13.743**	**10.389**	**2.106**	**12.052**	**3.047**	**0.387**
Precision (% RSD)	**17.582**	**11.907**	**9.667**	**5.691**	**9.735**	**6.058**
Day 2 Mean (*N* = 6)	0.119	0.237	0.546	0.639	1.057	2.502
SD	0.021	0.027	0.039	0.027	0.147	0.144
Accuracy (% error)	**19.194**	**5.204**	**9.123**	**14.785**	**5.736**	**0.069**
Precision (% RSD)	**17.446**	**11.294**	**7.085**	**4.277**	**13.949**	**5.745**
Day 3 Mean (*N* = 6)	0.102	0.230	0.497	0.803	0.968	2.499
SD	0.018	0.030	0.055	0.090	0.057	0.178
Accuracy (%error)	**2.485**	**8.070**	**0.622**	**7.103**	**3.172**	**0.030**
Precision (%RSD)	**17.454**	**13.132**	**11.123**	**11.216**	**5.882**	**7.108**

*Interday*						
Average concentration (*μ*g/mL)	0.112	0.248	0.518	0.701	1.019	2.504
SD	0.020	0.035	0.050	0.093	0.109	0.149
Accuracy (% error)	**11.807**	**0.962**	**3.536**	**6.578**	**1.870**	**0.142**
Precision (% RSD)	**17.667**	**14.174**	**9.659**	**13.289**	**10.657**	**5.948**

**Table 3:** Comparison of previously established methods for CS detection and quantitation.

Method	LOD (*μ*g/mL)	LOQ (*μ*g/mL)	Application	Shortcomings	References
Spectrophotometry	0.22	0.67	Drug mixtures, epicrom eye drops	Does not investigate selectivity regarding endogenous skin components, and LOQ is not ideal for lower limits of drug detection in permeation studies	El-Zahar et al. (2020) [26]
HPLC-UV	0.563	1.719	Nasal formulations and combined drug nasal preparations	Sensitivity and selectivity are not tested for drug detection in cutaneous permeation studies	Fathy et al. (2017) [1]
AUC and FDSFS methods	0.21 and 0.02	0.63 and 0.07	Aqueous solutions prepared with water, nazocrom nasal spray	FDSFS has sufficient LOD and LOQ, however does not investigate potential skin component interference	Abdel-Aziz et al. (2014) [31]
HPLC-UV	0.707	2.143	Aqueous solutions prepared from stock, fluca eye drops	Lacks sensitivity and does not evaluate potential skin interference	El-Bagary et al. (2016) [30]
Kinetic spectrophotometric method	0.0027	Not established	Diluted human serum and urine samples	Linearity was established up to 0.036 *μ*g/mL which would be too time and resource demanding for diluting permeation study samples and does not investigate interference from endogenous skin components	Keyvanfard et al. (2013) [41]
HPLC-UV	6.359	38.805	Stock solution, nasotal nasal spray	The method is not sensitive enough to detect smaller drug quantities associated with permeation studies, did not investigate skin interference	Hassib et al. (2011) [27]
Ion-paired HPLC with solid phase extraction	0.05	0.25	Human urine samples	Sensitivity seems appropriate; however, the method is time and resource intensive and does not investigate potential interference from skin	Aswania et al. (1997) [44]
HPTLC and HPLC	0.51 *μ*g/band and 0.129	0.17 *μ*g/band and 0.043	Drug samples in methanol, solutions of fluca eye drops or rabbit aqueous humor	Neither method evaluates overlap with endogenous biological components as rabbit aqueous humor was deproteinized before analysis, and investigation on interference from skin was not present	Hegazy et al. (2018) [32]
LC-UV	Not established	Not established	Nasal, inhaled, and ophthalmic solutions, inhaled powder	This method does not have enough evidence for sufficient selectivity or sensitivity for biological tissue/fluid analysis, did not investigate skin interference	Ng (1994) [33]
ELISA and GC/MS	<0.0006	0.009	Equine urine samples	Both methods are more time and resource demanding as ELISA assays requires sample dilution from 0.001 to 0.1 *μ*g/mL for optimal selectivity and GC/MS requires purification, evaporation, and derivatization of samples, neither were assessed for potential skin interference	Leavitt et al. (1993) [40]
Absorptive stripping voltammetry with HMDE	Not established	Not established	Drug spiked urine, 4% CS water solutions	Authors conclude that the method is ideal for trace detection, but the method is not accurate or precise enough for analyses where CS is the primary component, did not investigate skin interference	Moreira et al. (1992) [39]
Ion-paired HPLC	≤0.25	0.25	Drug solutions with 30 : 70 acetonitrile: water diluent	Ion-paired HPLC is time consuming and costly as previously described, did not investigate skin interference	Barnes et al. (2002) [28]
HPLC-UV	92	Not established	Nasal solution	The method established is more selective and proposed for the stability indication of CS impurities rather than CS quantitation, did not investigate skin interference	Mansfield et al. (1999) [29]
HPLC-UV	0.06	0.19	Drug solutions with mobile phase	Use of the method in selectively quantifying CS from other biological compounds or skin interference was not investigated	Segall et al. (1997) [34]
HPLC-MS/MS	0.0003	0.0003	Nasal drops and spray, human plasma	Linearity established up to 0.02 *μ*g/mL where permeation studies need to quantify higher drug contents and ESI+does not ionize CS as easily, did not investigate skin interference	Xu et al. (2008) [42]
HPLC-UV	0.05	0.05	Human urine samples with or without co-administered drugs	Method is sensitive but does not evaluate possible skin component interference and requires additional extraction and purification steps	Gardner (1984) [43]
HPTLC-UV densitometry	Not established	Not established	Drug solutions and gels, intal capsules, solutions, and gels	Linearity was validated at too small of a range for skin permeation studies and lacked analysis of skin interference	Kocic-pesic et al. (1992) [36]
HPLC-UV	Not established	Not established	Drug solutions and gels, intal capsules, solutions, and gels	Linearity was validated at too small of a range for skin permeation studies and lacked analysis of skin interference	Radulovic et al. (1994) [35]

## Data Availability

The data used to support the findings of this study are included within the article in the form of tables and figures. Additional information can be provided by the corresponding author as needed.

## References

[1] Fathy M. E., Abo El Abass Mohamed S., Elmansi H., Belal F. (2017). Simultaneous determination of cromolyn sodium combined dosage forms using isocratic HPLC method. *Journal of Chromatographic Science*.

[2] Rakesh R., Anoop K. R. (2012). Formulation and optimization of nano-sized ethosomes for enhanced transdermal delivery of cromolyn sodium. *Journal of Pharmacy & Bioallied Sciences*.

[3] Moore C., Ehlayel M. S., Junprasert J., Sorensen R. U. (1998). Topical sodium cromoglycate in the treatment of moderate-to-severe atopic dermatitis. *Annals of Allergy, Asthma & Immunology: Official Publication of the American College of Allergy, Asthma, & Immunology*.

[4] Murphy S., Kelly H. W. (1987). Cromolyn sodium: a review of mechanisms and clinical use in asthma. *Drug Intelligence and Clinical Pharmacy*.

[5] Puri A., Sivaraman A., Zhang W., Clark M. R., Banga A. K. (2017). Expanding the domain of drug delivery for HIV prevention: exploration of the transdermal route. *Critical Reviews in Therapeutic Drug Carrier Systems*.

[6] Ariyanayagam M., Barlow T. J., Graham P., Hall-Smith S. P., Harris J. M. (1985). Topical sodium cromoglycate in the management of atopic eczema-a controlled trial. *British Journal of Dermatology*.

[7] Angelo T., Pires F. Q., Gelfuso G. M., da Silva J. K. R., Gratieri T., Cunha-Filho M. S. S. (2016). Development and validation of a selective HPLC-UV method for thymol determination in skin permeation experiments. *Journal of Chromatography. B, Analytical Technologies in the Biomedical and Life Sciences*.

[8] Mansour R. S. H., Hamdan I. I., Salem M. S. H., Khalil E. A., Sallam A. A. (2021). HPLC method development/validation and skin diffusion study of caffeine, methyl paraben and butyl paraben as skin-diffusing model drugs. *PLoS One*.

[9] Tessema E. N., Gebre-Mariam T., Frolov A., Wohlrab J., Neubert R. H. H. (2018). Development and validation of LC/APCI-MS method for the quantification of oat ceramides in skin permeation studies. *Analytical and Bioanalytical Chemistry*.

[10] Bortolon F. F., Sato M. E., Andreazza R. C. D. S., Bresolin T. M. B. (2008). Effect of enhancers on the in vitro percutaneous absorption of piroxicam from compounding formulations. *Revista Brasileira de Ciências Farmacêuticas*.

[11] Liu X. Y., Qu T. T., Wang B. J. (2008). Determination of sodium cromoglycate in human plasma by liquid chromatography with tandem mass. *Biomedical Chromatography:Biomedical Chromatography*.

[12] Ozou M. L., Girault J., Malgouyat J. M., Pasquier O. (2001). Determination of sodium cromoglycate in human plasma by liquid chromatography-mass spectrometry in the turbo ion spray mode. *Journal of Chromatography - B: Biomedical Sciences and Applications*.

[13] Lin Z. J., Abbas R., Rusch L. M., Shum L. (2003). Development and validation of a sensitive liquid chromatographic-tandem mass spectrometric method for the determination of cromolyn sodium in human plasma. *Journal of chromatography. B, Analytical Technologies in the Biomedical and Life Sciences*.

[14] Li L. C., Vu N. T., Allen L. V. (1992). Iontophoretic permeation of sodium cromoglycate through synthetic membrane and excised hairless mouse skin. *Journal of Pharmacy and Pharmacology*.

[15] Tavano L., Nicoletta F. P., Picci N., Muzzalupo R. (2016). Cromolyn as surface active drug (surfadrug): effect of the self-association on diffusion and percutaneous permeation. *Colloids and Surfaces. B, Biointerfaces*.

[16] Gupta S. K., Kumar S., Bolton S., Behl C. R., Malick A. W. (1994). Optimization of iontophoretic transdermal delivery of a peptide and a non-peptide drug. *Journal of Controlled Release*.

[17] Gupta S. K., Kumar S., Bolton S., Behl C. R., Malick A. W. (1994). Effect of chemical enhancers and conducting gels on iontophoretic transdermal delivery of cromolyn sodium. *Journal of Controlled Release*.

[18] Nicoli S., Padula C., Aversa V. (2008). Characterization of rabbit ear skin as a skin model for in vitro transdermal permeation experiments: histology, lipid composition and permeability (abstract). *Skin Pharmacology and Physiology*.

[19] Ferry L. L., Argentieri G., Lochner D. H. (1995). The comparative histology of porcine and Guinea pig skin with respect to iontophoretic drug delivery. *Pharmaceutica Acta Helvetiae*.

[20] García-Alvarez-Coque M. C., Ramis-Ramos G., Ruiz-Angel M. J. (2019). *Encyclopedia of Analytical Science*.

[21] Puri A., Nguyen H. X., Banga A. K. (2016). Microneedle-mediated intradermal delivery of epigallocatechin-3-gallate. *International Journal of Cosmetic Science*.

[22] Guideline I. C. H. H. T. (2005). *Validation of Analytical Procedures: Text and Methodology Q2 (R1)*.

[23] Matteo C., Dovrtelova G., Di Clemente A. (2020). HPLC-MS/MS measurement of lidocaine in rat skin and plasma. application to study the release from medicated plaster. *Journal of Chromatography. B, Analytical Technologies in the Biomedical and Life Sciences*.

[24] Benson H. A. E., Watkinson A. C. (2012). *Transdermal and Topical Drug Delivery: Principles and Practice*.

[25] Patel R. R., Chaurasia S., Khan G., Chaubey P., Kumar N., Mishra B. (2016). Highly water-soluble mast cell stabilizer-encapsulated solid lipid nanoparticles with enhanced oral bioavailability. *Journal of Microencapsulation*.

[26] El Zahar N. M., Tadros M. M., Ayoub B. M. (2020). Development of advanced chemometric-assisted spectrophotometric methods for the determination of cromolyn sodium and its alkaline degradation products. *Molecules*.

[27] Hassib S. T., El-Zaher A. A., Fouad M. A. (2011). Development and validation of RP-HPLC stability-indicating methods for the determination of butamirate citrate and sodium cromoglycate. *Journal of Chemical and Pharmaceutical Research*.

[28] Barnes M., Mansfield R., Thatcher S. (2002). The selection of an ion pairing reagent for developing and validating a stability-indicating HPLC method for cromolyn sodium and its known impurities. *Journal of Liquid Chromatography & Related Technologies*.

[29] Mansfield R., Huang J., Thatcher S., Miller R. B., Davis C. W. (1999). Development and validation of a stability-indicating HPLC method for the determination of cromolyn sodium and its related substances in cromolyn sodium drug substance and cromolyn sodium inhalation solution, 1.0%. *Journal of Liquid Chromatography & Related Technologies*.

[30] El-Bagary R. I., Fouad M. A., El-Shal M. A., Tolba E. H. (2016). Stability-indicating RP-HPLC methods for the determination of fluorometholone in its mixtures with sodium cromoglycate and tetrahydrozoline hydrochloride. *Journal of Chromatographic Science*.

[31] Abdel-Aziz O., El-Kosasy A. M., Magdy N., El Zahar N. M. (2014). Novel spectroscopic methods for determination of cromolyn sodium and oxymetazoline hydrochloride in binary mixture. *Spectrochimica Acta. Part A, Molecular and Biomolecular Spectroscopy*.

[32] Hegazy M. A., Abdelwahab M. H., Hendawy H. A. M., Weshahy S. A., Abbas S. S. (2018). Validated HPTLC and HPLC methods for determination of fluorometholone and sodium cromoglycate in presence of their impurities and degradation products; application to kinetic study and on rabbit aqueous humor. *Journal of Liquid Chromatography & Related Technologies*.

[33] Ng L. L. (1994). Reversed-phase liquid chromatographic determination of cromolyn sodium in drug substance and select dosage forms. *Journal of AOAC International*.

[34] Segall A., Vitale F., Ricci R., Giancaspro G., Pizzorno M. T. (1997). High performance liquid chromatographic determination of sodium cromoglycate. *Drug Development and Industrial Pharmacy*.

[35] Radulovic D., Kocic-Pesic V., Pecanac D., Zivanovic L. (1994). HPLC determination of sodium cromoglycate in pharmaceutical dosage forms. *Farmaco*.

[36] Kocic-Pesic V., Radulovic D., Pecanac D., Zivanovic L. (1992). Determination of sodium cromoglycate in pharmaceutical dosage forms using TLC-densitometry. *Farmaco*.

[37] Cabral Marques H. M., Orth H., Schmidt P. C. (1999). Simultaneous determination of lactose and sodium cromoglycate in a dry powder inhalation formulation. *Die Pharmazie*.

[38] Huang T. M., Yang H., Liu Z., Sha Y. F., Duan G. L. (2005). Determination of the content of dexamethasone sodium phosphate and sodium cromoglycate by HPLC. *Fudan University Journal of Medical Sciences*.

[39] Moreira J. C., Foster S. E., Rodrigues J. A., Fogg A. G. (1992). Differential-pulse adsorptive stripping voltammetric determination of sodium cromoglycate at a hanging mercury drop electrode. *Analyst*.

[40] Leavitt R., Farmer W. H., Paterson P., Firby P. (1993). Sodium cromoglycate in race horses: development of enzyme-linked immunosorbent assay (elisa) screening and gas chromatography/mass spectrometry confirmation. *Canadian Society of Forensic Science Journal*.

[41] Keyvanfard M., Alizad K., Shakeri R. (2013). Determination of sodium cromoglycate by a new kinetic spectrophotometric method in biological samples. *Journal of Chemistry*.

[42] Xu X. Y., Zhang R., Yuan G. Y., Wang B. J., Liu X. Y., Guo R. C. (2008). HPLC-MS/MS method for determination of sodium cromoglycate concentration in human plasma and its pharmacokinetics. *Yaoxue Xuebao*.

[43] Gardner J. J. (1984). Determination of sodium cromoglycate in human urine by high-performance liquid chromatography on an anion-exchange column. *Journal of Chromatography*.

[44] Aswania O. A., Corlett S. A., Chrystyn H. (1997). Development and validation of an ion-pair liquid chromatographic method for the quantitation of sodium cromoglycate in urine following inhalation. *Journal of Chromatography - B: Biomedical Sciences and Applications*.

[45] Mulabagal V., Annaji M., Kurapati S. (2020). Stability-indicating HPLC method for acyclovir and lidocaine in topical formulations. *Biomedical Chromatography:Biomedical Chromatography*.

